# Improving the characterization of endothelial progenitor cell subsets by an optimized FACS protocol

**DOI:** 10.1371/journal.pone.0184895

**Published:** 2017-09-14

**Authors:** Karin Huizer, Dana A. M. Mustafa, J. Clarissa Spelt, Johan M. Kros, Andrea Sacchetti

**Affiliations:** Department of Pathology, Erasmus MC, Rotterdam, The Netherlands; European Institute of Oncology, ITALY

## Abstract

The characterization of circulating endothelial progenitor cells (EPCs) is fundamental to any study related to angiogenesis. Unfortunately, current literature lacks consistency in the definition of EPC subsets due to variations in isolation strategies and inconsistencies in the use of lineage markers. Here we address critical points in the identification of hematopoietic progenitor cells (HPCs), circulating endothelial cells (CECs), and culture-generated outgrowth endothelial cells (OECs) from blood samples of healthy adults (AB) and umbilical cord (UCB). Peripheral blood mononuclear cells (PBMCs) were enriched using a Ficoll-based gradient followed by an optimized staining and gating strategy to enrich for the target cells. Sorted EPC populations were subjected to RT-PCR for tracing the expression of markers beyond the limits of cell surface-based immunophenotyping. Using CD34, CD133 and c-kit staining, combined with FSC and SSC, we succeeded in the accurate and reproducible identification of four HPC subgroups and found significant differences in the respective populations in AB vs. UCB. Co-expression analysis of endothelial markers on HPCs revealed a complex pattern characterized by various subpopulations. CECs were identified by using CD34, KDR, CD45, and additional endothelial markers, and were subdivided according to their apoptotic state and expression of c-kit. Comparison of UCB-CECs vs. AB-CECs revealed significant differences in CD34 and KDR levels. OECs were grown from PBMC-fractions We found that viable c-kit^+^ CECs are a candidate circulating precursor for CECs. RT-PCR to angiogenic factors and receptors revealed that all EPC subsets expressed angiogenesis-related molecules. Taken together, the improvements in immunophenotyping and gating strategies resulted in accurate identification and comparison of better defined cell populations in a single procedure.

## Introduction

Over the last decades circulating EPCs have been extensively studied in the context of both health and disease. EPCs take part in neovascularization and their levels are used to monitor the effects of therapy [1–4]. Notably, the term EPC is not only used for cells with genuine endothelial lineages, but also for other cell types supporting neovascularization, including hematopoietic progenitor cells (HPCs) [1, 5–8]. HPCs are bone marrow derived [9] and home to ischemic or neoplastic tissues that secrete chemo-attractants and, following differentiation, contribute to angiogenesis by secreting proangiogenic factors [10–12]. Another subset of circulating EPCs is capable of generating *in vitro* outgrowth endothelial cells (OECs). The *in vivo* equivalent of OECs is believed to contribute to vascular regeneration [7, 13–17]. While most circulating endothelial cells (CECs) are damaged or apoptotic mature endothelial cells with no progenitor potential [18–21], there may well be a small CEC fraction of viable endothelial progenitors from which OECs can be grown. However, the kinship of CECs and OECS has not been proven, mainly because authors used unsorted PBMCs or PBMCs enriched for specific markers using magnetic beads, instead of FACS sorting [1, 7, 20, 22–26]. The accurate identification of EPC subsets, and their subdivision, is challenged by the low frequencies of these cells in the bloodstream, the different ways of their isolation, and the discrepant immuno-phenotypical definitions used [1, 5, 8, 23, 24, 27–31]. The introduction of validated procedures of isolation and work-up would greatly improve accurate comparisons of the various populations and literature data on the EPC subsets, and shed more light on the genuine source of OECs [7, 32].

Here we present a protocol for the accurate identification, characterization, and subdivision of HPCs, CECs and culture-derived OECs from peripheral blood samples of healthy adults (AB) and umbilical cord blood (UCB). The procedure includes the analysis of stem cell markers [32] and RT-PCR on sorted cells allows for the detection of markers beyond cell-surface expression. By following the procedures described we succeeded in demonstrating the similarities between OECs and CECs, suggestive of kinship between these populations. PCR analysis to the distinct EPC subsets and HUVECs for the detection of angiogenic factors and receptors revealed angiogenic capacities of all subsets.

## Material & methods

### Medical-ethical considerations

This study was approved by the Medical Ethics Committee of the Erasmus Medical Center, Rotterdam, The Netherlands (MEC-2011-313) and carried out in adherence to the Code of Conduct of the Federation of Medical Scientific Societies in the Netherlands (http://www.federa.org/codes-conduct).

### Blood samples and preparation

Eighteen samples of adult peripheral blood (24–40 ml) and 15 samples of umbilical cord blood (12 ml) were used for this study. The samples were collected in BD vacutainer EDTA tubes and stored at room temperature in the dark for ≤18 hours. Blood was then diluted 1:1 with PBS-0,5 mM EGTA, and PBMCs were isolated using Ficoll Paque plus (GE Healthcare).

### FACS analysis and sorting

**Fig 1:**
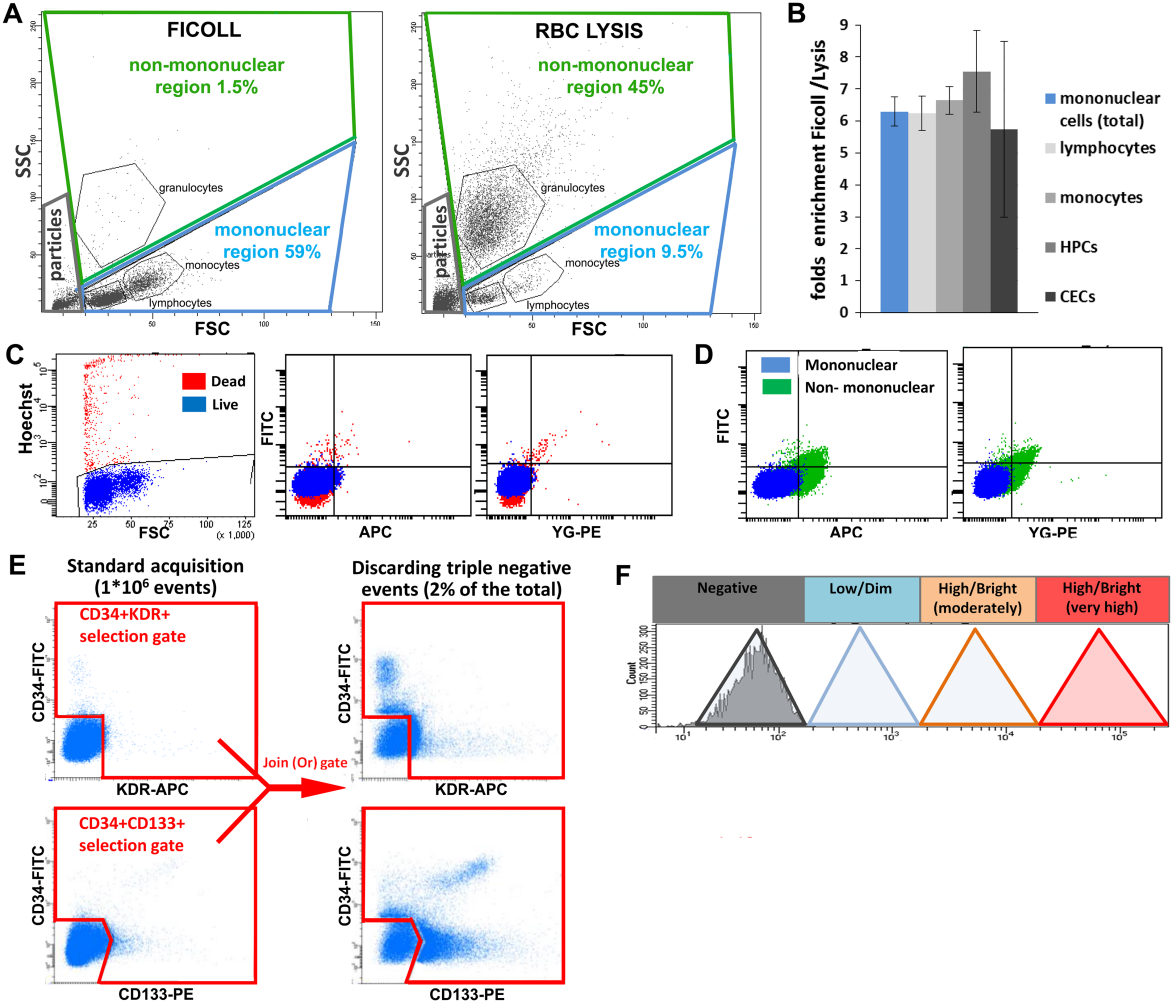
Basic strategies of sample preparation and FACS analysis. **A**. FACS plots of Ficoll-enriched PBMCs vs. RBC lysis-based preparations. To prevent exclusion of large cells, a large mononuclear-cell gate was applied. **B**. Ratios of enrichment of total live PBMCs, lymphocytes, monocytes, HPCs (as defined in Fig 2) and CECs (as defined in Fig 3) by Ficoll vs. RBC lysis. **C-D**. Examples of reduction of background (auto-fluorescence) by stringent exclusion of dead cells using Hoechst 33258 (C) and (residual) granulocytes (D). **E**. Basic procedure used, in addition to standard acquisition (1x10^6^ total events), to enrich for target cells by discarding triple CD34/CD133/KDR negative events. Upper left plot: selection of CD34^+^ and KDR^+^ cells; lower left plot: selection of CD34^+^ and CD133^+^ cells. Right panels: result of a “Join (Or) gate” in FACS DIVA. KDR^+^ CD34^+^ (upper plot) and CD133^+^ CD34^+^ (lower plot) populations are better visualized. **F**. Delineation of a scale of FACS-based fluorescence levels. Negative peak = unstained or isotype control; low (dim) = within 1 log from negative; high (bright) = more than 1 log from negative and ranging from moderately high (1^st^ to 2nd log above negative) to very high (3rd log or higher). “Medium” is sometimes used to define populations in between low and high.

PBMCs were incubated with 10% mouse serum to block unspecific antibody binding and stained 20’ with specific antibodies (S1 Table). To get saturation, OECs/HUVECs were stained with 1 μg Ab/10^6^ cells/200 μl, and PBMCs with 1.5 μg Ab/10^7^ cells/200 μl. KDR staining was amplified using a 3-step protocol: 1) anti-KDR-APC; 2) anti-APC-biotin; 3) streptavidin-APC. After staining the cells were washed twice and re-suspended in PBS, 10% BSA, 0,1μg/ml Hoechst 3h3258 to mark dead cells. All steps were performed on ice. Live nuclear staining was performed with the cell permeant Hoechst33342 (Sigma-Aldrich), 10μM for 30’ at RT. FACS analysis/sorting was performed with a BD FACS Aria III (BD Biosciences, New Jersey, US) using the parameters listed in (S1 Table). In the FSC/SSC plot, mononuclear cells were selected using a gate for high FSC cells excluding residual granulocytes, cellular debris and small particles (Fig 1 and S1 Fig). In the SSC-H/SSC-W and FSC-H/FSC-W plots single cells were selected and doublets excluded. Avital cells were gated out in a Hoechst-A/FSC-A plot. For the initial setup, fluorescence minus one (FMO) and isotype controls were used for each antibody (S2 Fig).

### Annexin V staining

Following immunostaining, 5μl of Annexin-V-FITC (BD) or Annexin-V-biotin (BD), were added to 500,000 PBMCs in Annexin Buffer and left 20’ on ice, followed by one washing step. Incubation with Annexin V-biotin was followed by streptavidin-APC staining. The FACS analysis was carried out in Annexin V buffer, 0,1μg/ml Hoechst 33258.

### Generation of outgrowth endothelial cells

PBMCs were re-suspended in endothelial cell medium (EGM-2 + BulletKit; Lonza), seeded in culture flasks (Corning, polystyrene) at 2.5x10^6^ cells/cm^2^, and incubated at 37°C, 5% CO2. Medium was changed daily. When OECs reached 80% confluence, cells were passaged using Accutase (Sigma-Aldrich). Gelatin, collagen-I, and fibronectin coating were tested and compared to non-coated plates. Because coating did not significantly affect the generation of OECs we used uncoated plates.

### RNA Isolation and RT-PCR

Gene expression was analyzed in HPCs (12 UCB and 10 AB; 2,000–70,000 cells), CECs (2 UCB and 1 AB; 600–2,000 cells), control leukocytes (3 UCB and 6 AB, CD34^-^CD133^-^KDR^-^CD45^+^), OECs (3 UCB and 1 AB; 500,000 cells), and HUVECs (2 separate cell lines, 500,000 cells). Cells were lysed in RLT buffer (Qiagen RNeasy micro kit) containing 1% β-mercaptoethanol, vortexed for 1’ and stored at -80°C. RNA was isolated using the RNeasy micro kit (Qiagen). cDNA was synthesized using the qScript cDNA SuperMix kit from Quanta. Due to low numbers of sorted HPCs and CECs, the PreAmp cDNA amplification kit (Quanta) was used (up to 100 genes). Amplified cDNA was diluted 20-fold. RT-PCR was performed following manufacturer instructions (Quanta) and 200nM primers. Pre-amplification was extensively validated to determine whether the correct proportion of transcripts was retained. PCR primers are listed in (S1 Table). In order to analyze the cells at functional level, RT-PCR to 10 angiogenic factors and receptors (apelin, PDFDβ, PDGFR, SCF, FGF, EGF, EGFR, VEGFA, Tie-1 and Tie-2) was carried out for all EPC subsets.

### Statistical analysis

The non-parametric Mann-Whitney U test in SPSS (version 21.0.0.1) was used to determine differences in HPC and CEC frequencies and in gene expression.

## Results

### Sample preparation and FACS analysis

The mononuclear cell fraction containing the EPC subsets was on average six times enriched by using Ficoll as compared to standard RBC lysis buffer, thereby leaving the ratios between the PBMCs and EPC subsets unaltered (Panels A and B in Fig 1 and Panel A and B in S1 Fig). The background caused by autofluorescence and unspecific antibody binding was reduced by excluding dead cells by Hoechst 33258 staining (Panel C in Fig 1) and residual non-mononuclear cells (Panel D in Fig 1) by gating them out in a FSC/SSC plot. The mononuclear gate applied was large enough to include CECs eventually present in the high FSC region (Panel A in Fig 1 and Panel C in S1 Fig). Spillover between fluorochromes was minimized by using one fluorescent channel per laser, with the exception of the 405 nm laser (S1 Table). Bright antibodies or signal amplification were used to gain sufficient resolution for each marker. The visualization of rare cells was improved by discarding triple CD34/KDR/CD133 negative events, thereby reducing data overload and enabling the recording of larger sized samples. By setting a threshold around 2% positive events, the target cells were ~ 50 times enriched (Panel E in Fig 1) and reliable visualization was accomplished for populations with a frequency as low as CECs in AB (~1/1*10^6^ PBMCs)[21]. To enable direct visual comparison of fluorescent levels between populations and samples, a common scale of fluorescence intensity based on literature and our own data was applied in all FACS plots (Panel F in Fig 1) [1, 33].

### Characterization and quantification of HPCs

**Fig 2:**
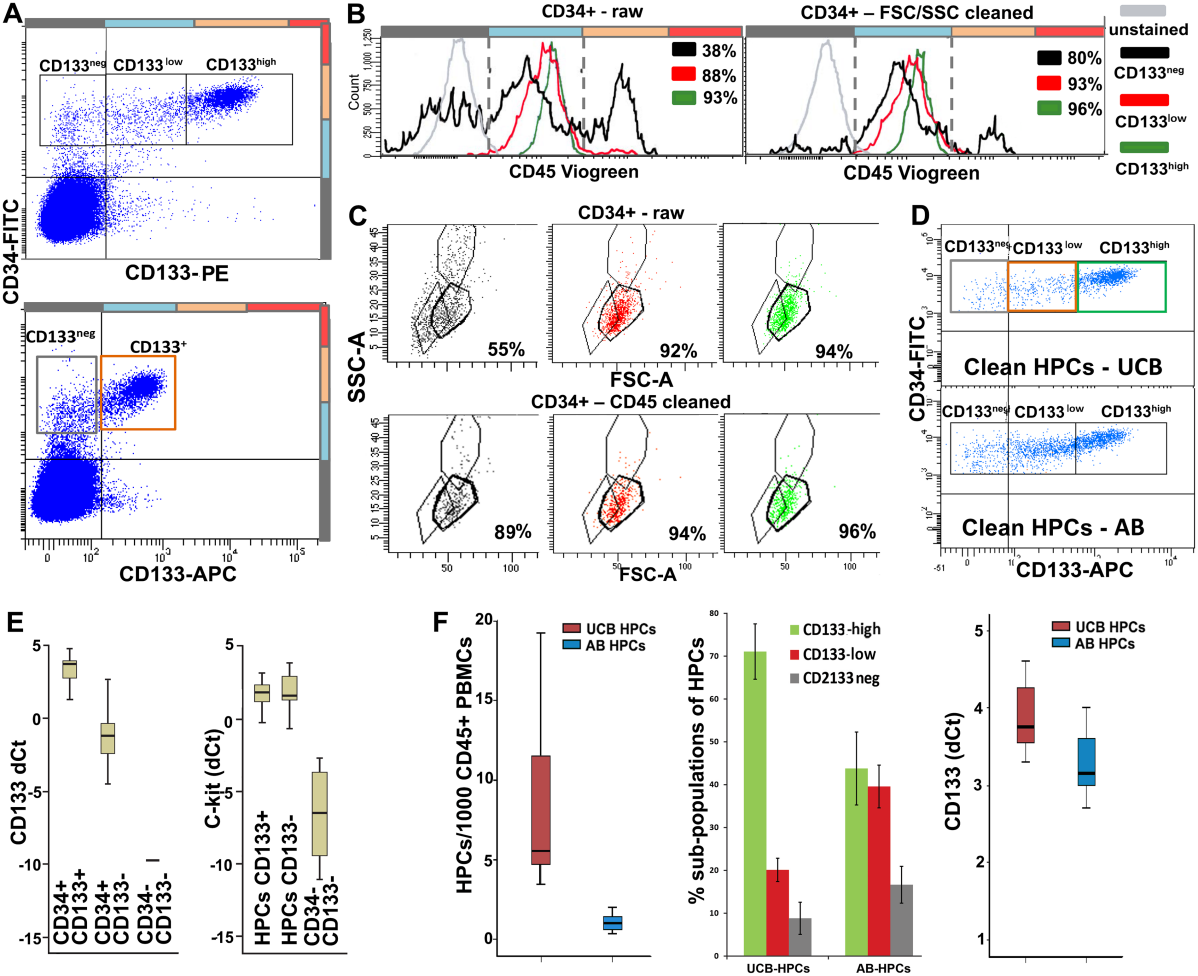
Identification and characterization of HPCs. **A**. Upper panel: HPCs in UCB and AB are initially selected as CD34^+/hi^ cells and subdivided into CD133^hi^, CD133^low^ and CD133^neg^. Lower panel: discrimination between CD133 levels is partially missed using a less bright antibody. **B**. HPCs are refined by gating a single peak in the CD45^low^ region. Left plot: CD45 gating on raw HPCs (CD34^+/hi^ events). Right plot: CD45 gating on CD34^+/hi^ events pre-refined by FSC/SSC. CD45 levels slightly increase with CD133 expression (see also S3 Fig), still remaining within the “low” gate. The % of match of raw or pre-refined HPCs with the CD45low gate is indicated in the plots for each population. **C**. In a FSC/SSC plot, HPCs appear as a tight cluster in between lymphocytes and monocytes. Upper panel: FSC/SSC gating on raw HPCs (CD34^+/hi^ events). Lower panel: FSC/SSC gating on CD34^+/hi^ events pre-refined by CD45 levels. The % of match of raw or pre-refined HPCs with the FSC/SSC gate is indicated in the plots for each population. Comparison of B (left vs. right plot) and C (upper vs. lower panel) shows that CD45 and FSC/SSC gating converge to the identification of pure HPCs: partial overlapping in HPC cleaning indicates that the two approaches identify the same population, however each approach also provides some independent contribution to HPC purification. The less pure and most affected by cleaning gates appears the CD133neg fraction. **D**. FACS plots showing the distribution of pure HPCs (CD34 vs. CD133 plot) in UCB vs. AB. **E**. CD133 and c-kit RT-PCR in CD34^+^CD133^+^ cells (n = 22); CD34^+^CD133^-^ cells (n = 17) and CD34^-^KDR^-^CD133^-^ PBMCs (n = 8) isolated from UCB. CD133 mRNA is detected in both CD133^+^ and CD133^-^ HPCs, although at different levels. c-kit mRNA further confirms the HPC identity of the CD34^+^CD133^-^ cells. **F**. Left panel: median frequency of HPCs in UCB (5,6 in 1*10^3^ CD45^+^ PBMCs) and AB (0,97 in 1*10^3^ CD45^+^ PBMCs, significantly lower than in UCB, Z = -4,9; p = 1,00E^-06^). Middle panel: percentage ± SD of the three CD133-based HPC subpopulations in UCB vs. AB. Right panel: RT-PCR confirms higher CD133 expression by total UCB-HPCs than AB-HPCs.

Following current definitions [5, 17, 34], HPCs were initially identified as a CD34^+/high^ cluster and refined by gating CD45^low^. Based on the expression intensities for CD133, HPCs were sub-divided in CD133^high^, CD133^low^, and CD133^neg^ cells (Panela A and B in Fig 2) [1, 5, 17, 34]. A bright antibody was necessary to properly visualize different CD133 levels (Panel A in Fig 2). HPCs were located in between lymphocytes and monocytes in the FSC/SSC plot (Panel C in Fig 2). Gating on FSC/SSC converged with CD45-based gating, and provided further purification of HPCs, mainly by cleaning the CD133^neg^ subpopulation. By using both CD45 and FSC/SSC, HPC gating was refined over previous protocols. To verify the identity of the HPCs we tested the expression of CD133 and c-kit by RT-PCR on sorted HPC-fractions. The expression of CD133 was verified by RT-PCR and was at low levels also found in the CD133^neg^ subpopulation (Panel E in Fig 2). mRNA expression for c-kit was found in all three subpopulations (Panel E in Fig 2) and appeared to be high and independent of CD133 levels. Overall, HPCs (calculated as ratio over CD45^+^ PBMCs) were 5.8 times more frequent in UCB than in AB (Panel F in Fig 2). The frequencies of the three CD133-based subpopulations differed between UCB and AB. CD133^high^ HPCs represented 72% of total HPCs in UCB but only 43% in AB.

**Fig 3:**
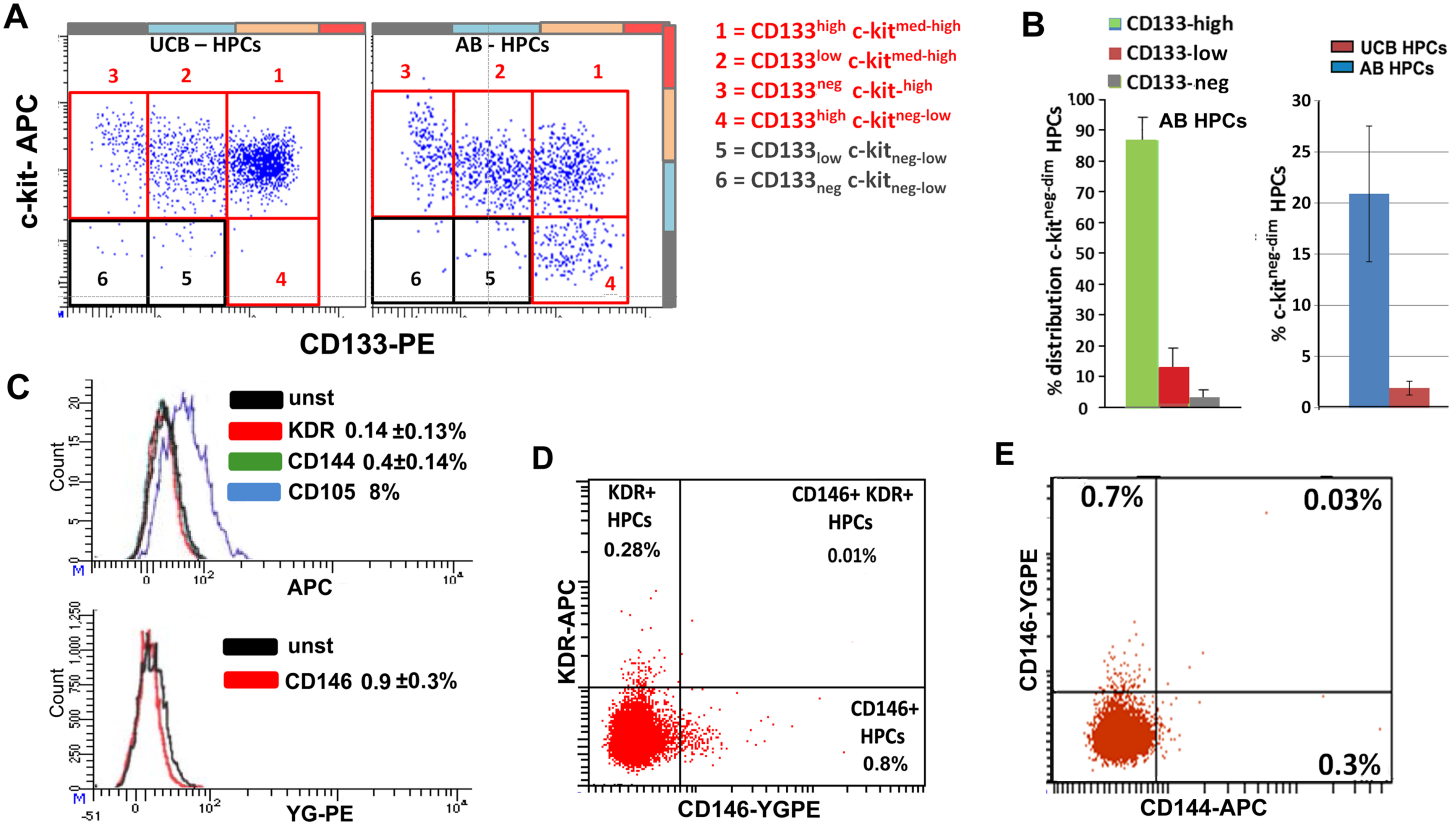
HPCs: Analysis of c-kit and endothelial markers. **A**. FACS analysis for c-kit vs. CD133 confirms the PCR results and refines HPC subdivision. In UCB, c-kit medium-high levels were observed in all the CD133-based subpopulations (with the exception of sporadic events). In AB, an additional c-kit^neg-low^ cluster was observed within the CD133^high^ subpopulation, while the highest c-kit levels were observed on the CD133^neg^ cells (see also S3 Fig). **B**. Left: distribution of c-kit^neg-low^ cells between the CD133-based subpopulations in AB. Right: quantification of c c-kit^neg-low^ cells in AB vs UCB. Right:. **C**. KDR, CD144, CD146 are sporadically expressed by HPCs. CD105 is dimly expressed but only a fraction of cells crosses the negative gate (8%). The percentages of positive cells are indicated in the plots. **D**. Analysis of KDR vs. CD146 expression in HPCs (UCB): the markers do not match but identify independent subpopulations. For better visualization, we used a UCB sample with high frequency of HPCs and relatively high KDR expression on HPCS (see also Panels D and E in S3 Fig). **E**. Analysis of CD144 vs. CD146 expression in HPCs: the two markers mostly identify independent subpopulations (see also Panel F in S3 Fig).

Further HPC sub-classification was obtained by simultaneous staining for c-kit and CD133. Four sub-populations of HPCs were distinguished: CD133^neg^c-kit^high^, CD133^low^c-kit^high^, CD133^high^c-kit^high^, and CD133^high^c-kit^neg/low^ (the last only observed in AB samples) (Panels A and B in Fig 3). In addition, the expression of CD34 and CD45 was found to be positively associated with CD133 levels but independent of c-kit levels. CD133^neg^ HPCs have the highest c-kit levels (Panel A in Fig 3 and Panels A-C in S3 Fig). Expression of the endothelial markers KDR, CD144, and CD146 was only observed in sporadic HPCs (Panels C-E in Fig 3). CD105 was expressed at very low levels, only partially crossing the negative gate. Preliminary co-expression analysis revealed that KDR, CD146, CD144, CD105 do not converge to the identification of a single population of EPCs [8], but each marker mostly identifies small independent subpopulations, (Panels D and E in Fig 3 and Panels D-F in S3 Fig).

### Characterization and quantification of CECs

**Fig 4:**
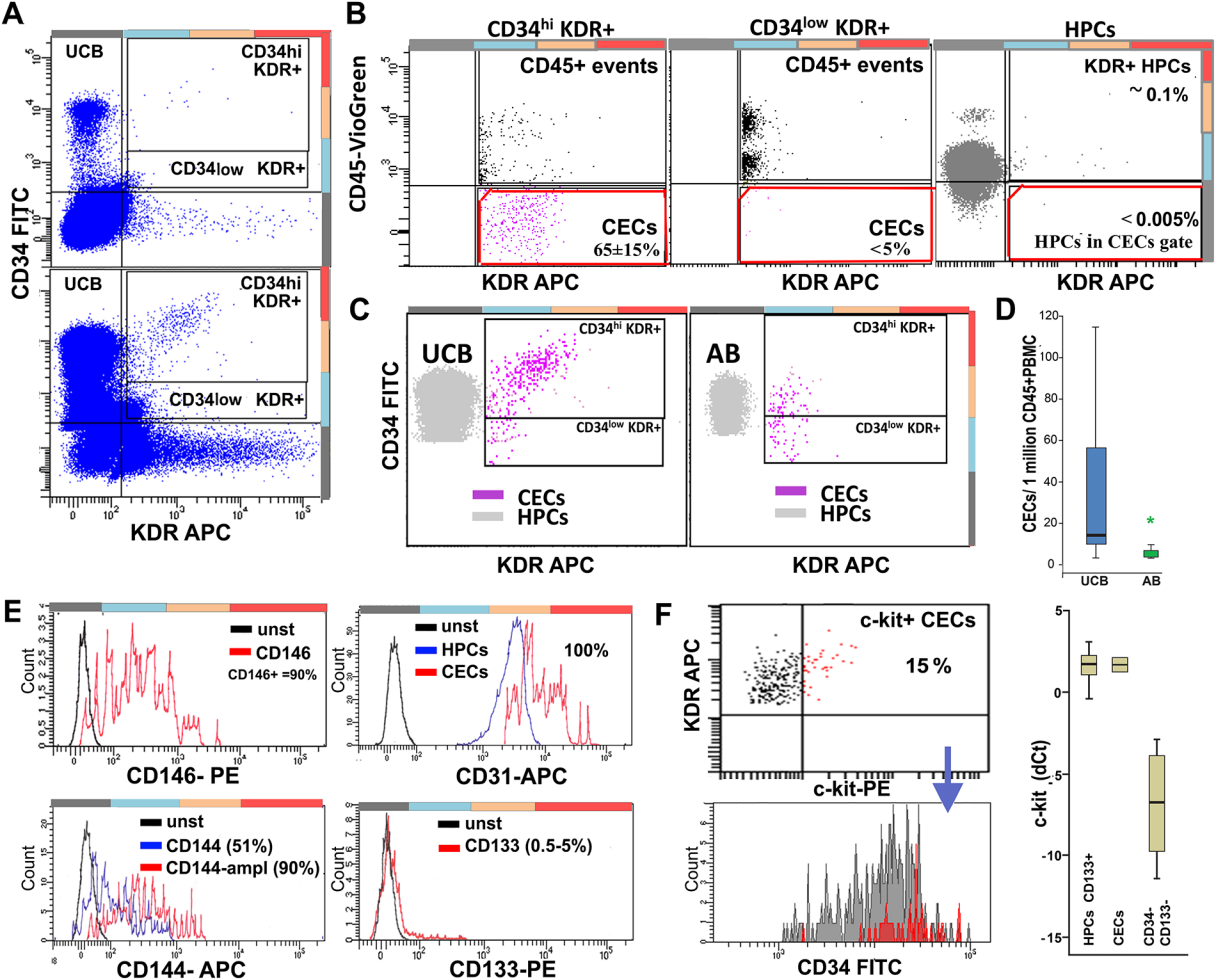
Identification, characterization, and quantification of CECs. **A**. Initial selection of CECs (UCB) on a CD34 vs. KDR plot (1x10^6^ events) by gating KDR^+^ cells at different CD34 levels. Lower panel: the acquisition of large sized samples was reached by discarding triple CD34/CD133/KDR negative events resulting in better visualization of CECs. **B**. Subsequently, true KDR^+^CD45^neg^ CECs are selected in a CD45 vs. KDR plot. Most CECs belong to the CD34 high region (left plot), since KDR^+^ CD34^low^ events are almost entirely CD45^+^ (middle plot). Right plot: the CD45 gate applied efficiently discriminates CECs from sporadic KDR^+^ HPCs (selected by CD34 and FSC-SSC). **C**. Comparison of UCB-CECs (left plot) and AB-CECs (right plot). For reference: HPCs are shown in gray. In UCB, most CECs cluster at CD34 levels above HPCs and are KDR^med-hi^, while in AB CECs have significantly lower CD34 and KDR levels. **D**. Frequency of CECs in UCB (14/10^6^ CD45^+^ PBMCs) vs. AB (1,9/10^6^CD45^+^ PBMCs). In AB the levels are significantly lower (Z = -3,6; p = 3,00E-04). **E**. Marker expression by CECs. Upper left: 90% of KDR-selected CECs express CD146. **Lower left**: 90% of CECs express CD144 but only after signal amplification. **Upper right**: CD31 is highly positive in CECs, but does not discriminate CECs from HPCs. **Lower right**: CD133 is expressed by a minority of CECs. **F. Upper left**: Around 15% of the CECs appear positive for c-kit and c-kit positivity correlates with higher KDR and CD34 levels (lower left). **Right panel**: RT-PCR confirms the expression of c-kit by CECs at levels comparable with HPCs (n = 3) despite significantly lower surface expression.

CECs were identified as CD34^+^KDR^+^ cells (Panel A in Fig 4) that are negative for CD45 (Panel B in Fig 4). Brightnesss of the staining was essential for proper CEC identification. Visualization of KDR required signal amplification (S1 Table) and the staining intensity of CD45 was crucial for separating CD45^neg^ CECs from sporadic CD45^low^KDR^+^ HPCs across the CD45^neg^ gate (Panel B in Fig 4). The frequency of CECs was significantly higher in UCB (14/10^6^ CD45^+^ PBMCs) than in AB (1,9/10^6^ CD45^+^ PBMCs) (Panels C and D in Fig 4). The CD34 and KDR levels encountered in UCB-CECs (mainly CD34^high^KDR^+/high^) differed from those in AB-CECs (mainly CD34^med^KDR^low^) (Panel C in Fig 4). The CECs expressed the endothelial markers CD146, CD144 and CD105 (Panel E in Fig 4 and Panel B in S4 Fig), which largely overlapped with KDR, confirming the endothelial identity of the selected population. Based on our data, CD146 and CD144 can be regarded as good substitutes for KDR in CEC identification, or as additional markers to refine the population. Immunopositivity for CD105 should be carefully evaluated since CD105-expressing HPCs (Panel C in Fig 3) that pass the CD45 negative gate may contaminate the CEC population. Notably, KDR yielded the cleanest background on HPCs followed by CD144, and CD146 (Panel C in Fig 3), and the best match (98% vs. only 90% with CD146 and CD144) in CEC-identification (Panel E in Fig 4 and Panel B in S4 Fig). RT-PCR confirmed that KDR expression is CEC-specific and absent in HPCs (Panel C in S4 Fig). Because CD31 was expressed at high levels in other cell types, including HPCs, it cannot be considered as a selective marker for CECs. Since CD133 was found to be expressed by few CECs it cannot be regarded as a negative marker for these cells (Panel E in Fig 4). Further subdivision of CECs was obtained by the analysis of c-kit. Around 15% of CECs appeared to be c-kit positive and c-kit expression was associated with higher CD34 and KDR levels. RT-PCR confirmed c-kit expression in CECs. Notably, c-kit mRNA levels were high and comparable to those of HPCs, despite much lower surface protein expression (Panel F in Fig 4), indicative of a discrepancy between mRNA and protein levels or surface expression of this marker.

**Fig 5:**
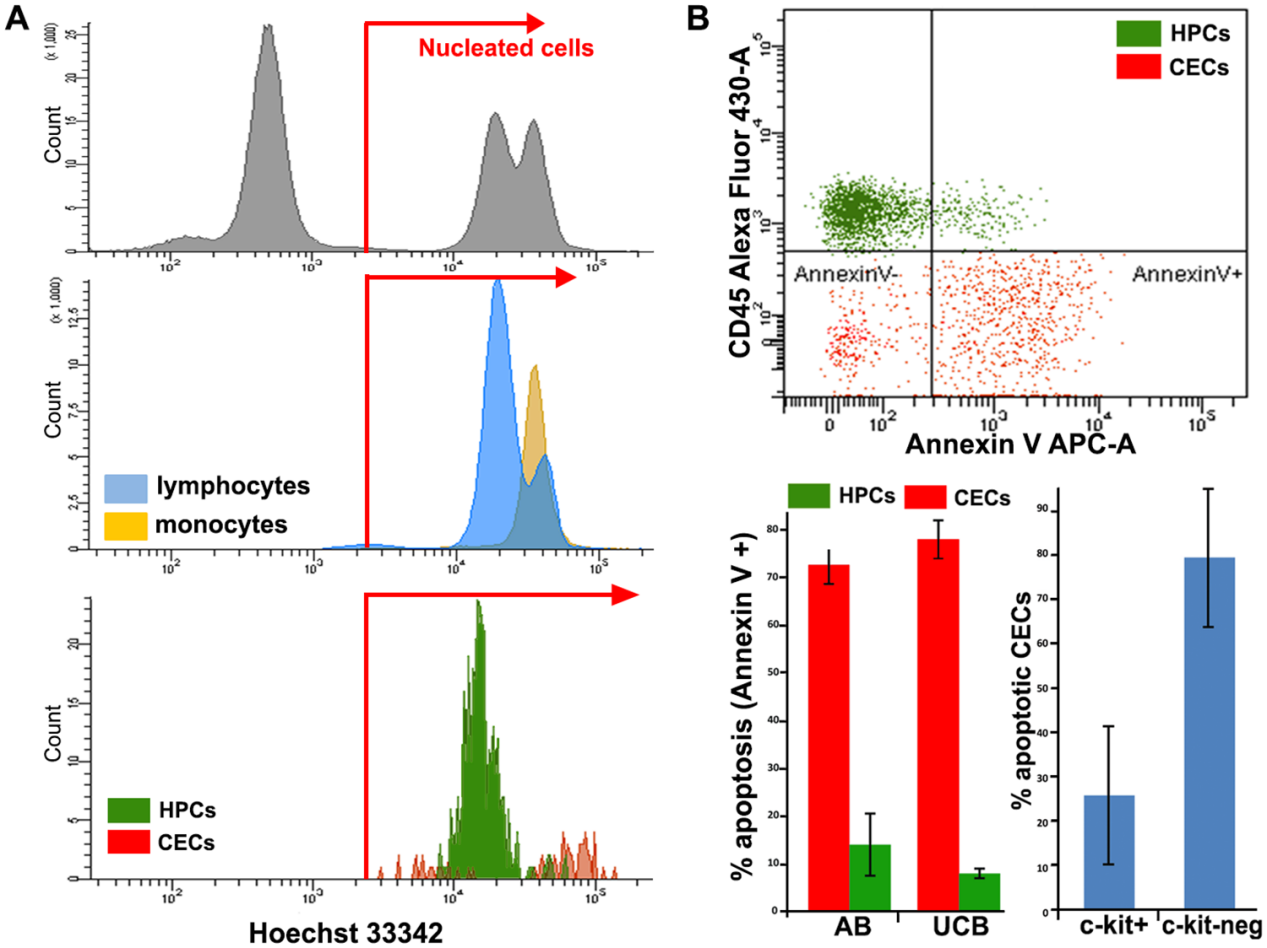
Definition of nucleated events and apoptosis in CECs and HPCs. **A**. FACS analysis of live nuclear staining with Hoechst 33342. Upper plot: nucleated events are separated from non-nucleated ones using erythrocytes and mononuclear cells as a reference. Medium plot: lymphocytes and monocytes are 100% nucleated. Lower plot: both CECs (red) and HPCs (green) appear as nucleated cells. CECs show a bimodal distribution with a sub-G1 fraction (probably apoptotic, DNA-fragmented cells). **B**. Upper plot: representative Annexin V staining of CECs (selected using CD34-FITC, CD146-PE, CD45-VioGreen) and HPCs (selected using CD34-FITC and CD45-VioGreen) from UCB. Bottom left: percentages of apoptotic CECs and HPCs in AB and UCB. Bottom right: apoptosis in c-kit+ vs c-kit^neg^ CECs.

In order to distinguish nucleated CECs from contaminating non-nucleated events, mainly aggregates of endothelial micro-particles, representative samples were stained with the nuclear dye Hoechst 33342 and all CECs appeared to be nucleated by FACS analysis (Panel A in Fig 5) and post-sorting microscopic inspection. Since CECs are reported to be apoptotic, apoptosis in these cells was assessed by Annexin-V staining. 78 (±4) % of CECs appeared to be apoptotic vs. only 8% of HPCs (Panel B in Fig 5). The pan-caspase inhibitor Z-VAD-FMK, added immediately after blood sampling, did not reduce the apoptotic fraction, which remained on average 80%, indicating that the cells were apoptotic from the time they entered the bloodstream. The expression of c-kit in CECs was associated with lower apoptosis (Panel B in Fig 5).

### Generation and characterization of OECs

**Fig 6:**
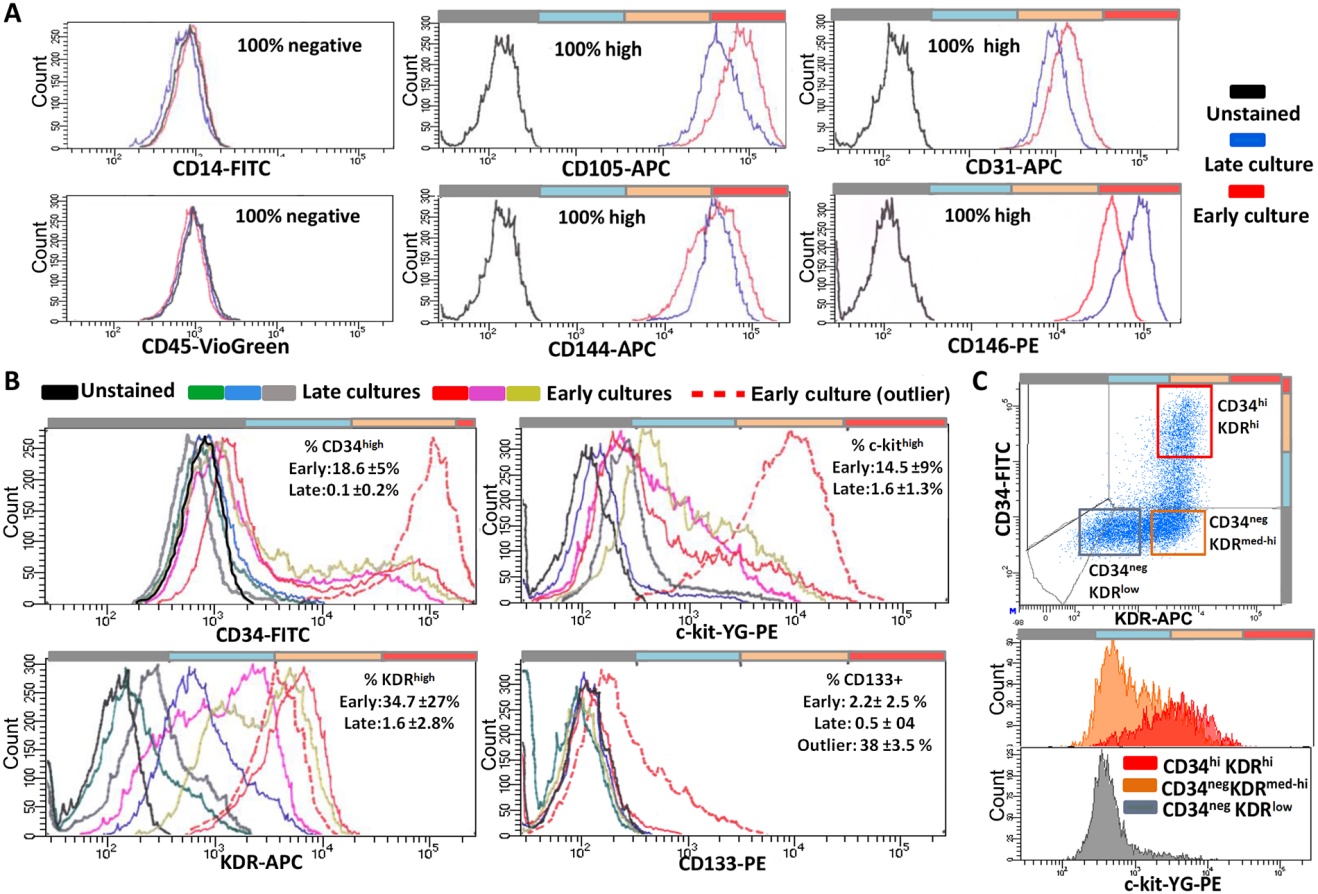
FACS analysis of OECs. FACS analysis of markers with homogeneous/stable **(A**) and heterogeneous/unstable (**B-C**) expression in early (passage 2–3) and late (passage >6) OEC cultures. **A**. No signals for CD45 and CD14. Staining for CD105, CD31, CD144, CD146 was homogeneously high and stable. **B**. CD34, KDR, and c-kit expression was heterogeneous and decreased with ageing of the cultures. The expression of CD133 was sporadic, with the exception of a single outlier, and slightly decreased with time in culture. For each marker, three examples of early and late cultures, and the outlier, are shown. **C**. Example of positive correlation (early passage OECs) between CD34, KDR, and c-kit. The markers define a triple high cluster, which is lost with ageing of the culture.

Following 2–10 days after seeding unsorted PBMCs, adherent spindle-shaped cells matching the morphologic characteristics of “early EPCs” (eEPCs) [1, 35–37] appeared in both AB and UCB at a similar frequency (Panels A and B in S5 Fig). Following 1–3 weeks of culturing, OECs or “late EPCs” appeared in 5 out of 6 UCB samples and 2 out of 10 AB samples (Panel C in S5 Fig). From UCB also more colonies were generated (20 to 30) than from AB (1 and 10) per PBMC sample corresponding to 10 ml of original blood. The OECs displayed the characteristic cobblestone morphology, expressed von Willebrand factor (S5 Fig) and formed tube-like structures after several days. The OECs were harvested and FACS analyzed. HUVECs were used as reference endothelial cells. Both OECs and HUVECs expressed high and stable levels of CD31, CD144, CD146 and CD105 while CD14 and CD45 were not expressed (Panel A in Fig 6 and Panel A in S6 Fig). With the exception of a single outlier, CD133 expression was restricted to ≤5% of the OECs (Panel B in Fig 6), matching the CD133 expression in CECs (Panel E in Fig 4). The levels of c-kit, KDR and CD34 were heterogeneous and correlated positively with each other (Panels B and C in Fig 6 and Panels B and C in S6 Fig). Early passage OECs and HUVECs contained a larger fraction of cells expressing high levels of CD34, KDR and c-kit in comparison to the late passage ones (Panel B in Fig 6 and Panels B and D in S6 Fig).

To investigate whether CECs are the source of OECs, we cultured sorted CECs in parallel with sorted and unsorted total PBMCs, using a larger nozzle (100μm) and lower pressure (20 psi) to reduce cell damage. The sorted cells (both CECs and total PBMCs) did not generate OECs, while unsorted PBMCs did. However, spindle-shaped early EPCs were successfully obtained from sorted CD14^+^ cells (Panel B in S5 Fig), or total sorted PBMCs. This confirmed the sound procedure of sample preparation and sorting, and suggested that FACS sorting is too damaging for the vulnerable OEC precursors to generate OECs in culture. No correlation between the yield of OECs and that of eEPCs was noticed in individual samples or in UCB vs. AB (S5 Fig), confirming that these two cell types have different circulating precursors [7, 22, 25, 36, 37].

### Functional analysis

We analyzed a total of 10 angiogenic factors and receptors (apelin, PDGFβ, PDGFR, SCF, FGF, EGF, EGFR, VEGFA, Tie-1 and Tie-2) by RT-PCR to all distinct EPC subsets and HUVECs. Angiogenic factors appeared to be expressed in all EPC subsets. However, there was variation in the levels of expression of these factors between the various EPC subsets (S7 Fig). The levels of all angiogenic factors and receptors were lowest in HPCs with the exception of EGF. Notably, CECs showed the highest expression of Apelin, PDGFβ, SCF, EGFR, and high expression of Tie-2 as compared to the other cell types. The expressional pattern of OECs and HUVECs almost completely overlapped. Overall, the pattern of OECs was much closer to CECs than to HPCs.

In addition, we added immunohistochemistry for endothelial markers to OECs (vWF, CD31, CD105) (S8 Fig). OECs were positive for all these markers, indicative of their endothelial lineage.

## Discussion

In this study we describe a procedure that allows for efficient identification, quantification, and sorting of HPCs and CECs from peripheral blood samples, and culture-generated OECs. Moreover, we improve the characterization of these populations resulting in, for instance, better definition of subpopulations, and identification of a candidate circulating OEC precursor. By using a Ficoll-based density gradient instead of a standard RBC lysis, significant enrichment for PBMCs was achieved with preservation of the relative frequencies of HPCs and CECs over total PBMCs [38, 39]. Ficoll-enrichment, combined with stringent gating out of residual non-mononuclear cells, dead cells and debris, and accurate selection of markers, yielded clean and pure populations meeting the current standards of state-of-the art FACS procedures [40]. The procedure significantly reduced the sample size with preservation of the relative frequencies of HPCs and CECs over total PBMCs, allowing for significantly faster analysis and sorting of the target cells. The visualization and quantification of small populations was improved by selecting the events that were positive for the markers of interest to reduce data overload. This allowed recording the equivalent of ~50*10^6^ CD45^+^ PBMCs within 1*10^6^ events (about 10–25 ml of whole blood). Moreover, by avoiding the use of toxic nuclear dyes the cells remain fully viable for subsequent culture experiments and RNA extraction. By using the outlined protocol we were able to identify HPCs and CECs more accurately, and isolate 200 to30.000 cells for RT-PCR analysis from rare populations with frequencies as low as 1/1*10^6^ CD45^+^ PBMCs.

### Improved characterization of HPCs

The current definition of HPCs is mainly based on a CD34^+/high^CD45^low^ signature with CD133 expression as an additional marker [5, 17, 34]. In the present analysis HPCs were identified as a CD34^+/high^ CD45^low^ cluster and further cleaned by gating a specific region in FSC/SSC (Panels B and C in Fig 2). Moreover, extending the definition of CD133^+^ and CD133^neg^ HPCs [17, 34], we propose a subdivision of HPCs in CD133^high^, CD133^low^, and CD133^neg^ subpopulations. The HPC-identity of these subpopulations was confirmed by RT-PCR for CD133 and c-kit. By means of additional c-kit staining, HPCs were further subdivided by the demonstration of a c-kit^neg/low^ subpopulation within the CD133^high^ HPCs that was found only in AB, not UCB. So far, c-kit^neg/low^ HPCs have not been identified in human blood samples and were only described as a quiescent population in mice [41]. CD133 or c-kit alone are no markers for the identification of HPCs [42, 43], since they range from negative to high. However, our data show that HPCs cannot be negative for CD133 and c-kit at the same time. Simultaneous staining of CD34+ cells (common denominator) for CD133 and c-kit leads to very accurate identification of HPCs as CD133^+^ and/or c-kit^+^ (Panels A and B in Fig 3). Following CD34, CD133, and-c-kit-based identification, FSC/SSC and CD45 gating can be applied to further purify the HPCs, in particular to exclude residual doublets with other cell types, and other false positives, mainly contaminating CECs.

In line with previous data [1, 27, 44], the frequency of HPCs was significantly higher in UCB than in AB. We observed a prevalence of CD133^bright^ HPCs in UCB vs. AB. This finding fits with the notion that CD133^high^ HPCs are more primitive stem cells serving functions in fetal and post-natal development [43, 45, 46]. We also found positive correlation of CD45 and CD34 expression with CD133 levels, and a negative correlation between c-kit and CD133 levels (S3 Fig). Analysis of the endothelial markers KDR, CD146, CD144, CD105 and CD31 lead to an estimation of the risk of HPC contamination of the CEC population using each single marker. In addition, the endothelial markers highlighted the presence of potentially interesting and rare subpopulations of HPCs like the KDR^+^HPCs subpopulation that was described by Case et al. [5], and considered as a distinct EPC population by other authors [1, 8, 27–30]. We show that the percentage of HPCs expressing endothelial markers (KDR, CD146, CD144, CD105) varies significantly and depends on the marker used. We also show that, with the exception of a few sporadic events, the markers are not concurrently expressed (Panels D and E in Fig 3 and Panels D-F in S3 Fig). Therefore, these HPCs expressing endothelial markers represent heterogeneous subpopulations requiring further exploration. Taken together, better identification of HPCs over the previous CD34^+/high^CD45^dim^ based protocol was reached and better subdivision was achieved by means of a combined CD34, CD133, and c-kit staining.

### Identification and characterization of CECs

In addition to the current criteria used for CEC-identification, we aimed at a more precise characterization of these cells, delineation of sub-populations, and sorting for RT-PCR and culture. Since CECs are generally identified as nucleated CD45^neg^CD34^+^ events that additionally express an endothelial marker, i.e., CD146 or KDR [18, 20, 21, 23], we used the CD34^+/high^ KDR^+^CD45^-^ profile as a common denominator (Panels A and B in Fig 4). KDR-based CEC identification was confirmed by CD146, CD105 and CD144 staining and by RT-PCR for these markers (Panel E in Fig 4 and Panel C in S4 Fig). Since a single coherent population of CECs was defined by using the endothelial markers, they may substitute KDR in the identification CECs. However, staining for KDR yields the lowest background on HPCs and the best cross-matching score and therefore, is regarded as a marker of first choice, followed by CD146 and CD144 (Panel E in Fig 4 and Panels A and B in S4 Fig). The quality of CD45 staining/gating was crucial for discriminating CECs from contaminating cells, mainly CD45^low^HPCs sporadically expressing KDR, CD146, CD144, and potentially crossing the CD45^neg^ gate (Panel B in Fig 4 and Panel A in S4 Fig). Notably, all the CECs identified by the current procedure were nucleated (Panel A in Fig 5), i.e. not contaminated by (endothelial) micro-particle aggregates and therefore, the use of cell-permeant nuclear dyes like Hoechst or DRAQ5 was not necessary [20, 47, 48]. By avoiding the use of bright and toxic nuclear dyes a fluorescent channel is saved and spillover to other channels minimized, thereby improving the capacity of the detection channels and the sensitivity of the analysis. Moreover, the viability of the cells is well preserved for subsequent culture and RNA extraction.

Besides confirming higher frequency of CECs in UCB vs. AB [1, 7, 21, 22, 32, 44], we found a major difference between CECs in AB as compared to UCB. The CECs in UCB express significantly higher levels of CD34 and KDR (Panel C in Fig 4). Moreover, by means of Annexin-V staining we identified two main subsets of CECs: a viable fraction of ~20%, and an apoptotic fraction of ~80%, equally present in AB and UCB (Panel B in Fig 5). In addition, a c-kit^+^ subset, expressing higher levels of KDR and CD34, was identified and found to be mainly present in the viable fraction of CECs (Panel F in Fig 4 and Panel B in Fig 5) This subset may well contain OEC-precursor cells. The angiogenic capacities of the CECs were further strengthened by the expression of angiogenic factors (S7 Fig).

### Characterization of OECs and prediction of their circulating precursor

The characterization of OECs was improved by various combinations of markers. In addition, comparing early vs. late cultures evidenced the alterations occurring with aging of the cultures and led to the definition of homogeneously high and stable markers (CD146, CD144, CD105, CD31) on the one hand, and heterogeneous and unstable ones on the other (KDR, CD34, c-kit, CD133). The comparisons also enabled us to trace back the OEC precursors to c-kit+ CECs. By combining FACS populations and RT-PCR data we found overlap in the expression of KDR, CD146, CD105, CD144, and CD31 by OECs/HUVECs and CECs. Surface expression of the markers was generally lower in CECs than in OECs/HUVECs. This may be explained by the fact that OECs/HUVECs are derived from *in vitro* cultures, while CECs are *in vivo* single circulating cells, exposed to different environmental stimuli (lacking, for instance, the adhesion to other endothelial cells, and exposed to the blood microenvironment) and subjected to a potentially stressful purification procedure. Interestingly, the stem cell-marker c-kit was expressed by a significant fraction of early passage OECs and HUVECs and positively associated with KDR and CD34 levels. CD34^high^KDR^high^c-kit^high^ cells decreased with passaging in culture (Panels B and C in Fig 5 and Panels B-D in S6 Fig). This finding points to a triple +/high phenotype (CD34^+/high^KDR^+/high^c-kit^+/high^), which is lost with aging in culture, as the progenitor/founder of both cell types [24, 49]. This view is supported by the recently described c-kit^+^KDR^+^CD105^+^CD45^-^ 'vascular endothelial stem cells’ (VESCs) in the blood vessels of adult mice, which are capable of many cycles of replication and to generate functional blood vessels *in vitro* [50]. Notably, the CD34^+/high^KDR^+/high^c-kit^+/high^ phenotype that we trace back as potential OEC precursor well matches the viable fraction of CECs expressing c-kit and higher levels of KDR and CD34 (Panel F in Fig 4 and Panel B in Fig 5).

The current opinion on the origin of CECs is that these cells are mostly apoptotic, mature endothelial cells without progenitor potential, shed from damaged blood vessels [14, 30, 51]. The present data, however, show that about 20% of circulating CECs are viable and express c-kit (Panel F in Fig 4 and Panel B in Fig 5). The high expression of angiogenic factors by CECs confirmed that these cells contain a fraction potentially contributing to neovascularization and *in vitro* generation of OECs. Unfortunately, we were unable to directly derive OECs from FACS-sorted CECs. These results are in line with results explicitly published by others [23] and with the (implicit) evidence that OECs are reported to be generated from unsorted or immuno-magnetic-bead-sorted PBMC fractions, not FACS-sorted ones [1, 7, 22–26]. The present data, pointing to viable CECs expressing c-kit as OEC precursors, are in line with data obtained from cells purified by antibody-coated beads suggesting that the OEC-founder cells do not originate from bone marrow and express CD34 and CD146, but not CD45 or CD133, a pattern compatible with CECs [7, 22, 23]. The significant higher yield of OECs from UCB (containing more CECs than AB) than from AB further corroborates the view that OECs stem from CECs.

## Conclusions

In conclusion, the present FACS-based analysis yields optimal identification, characterization and sub-division of cell populations meeting the current criteria for HPCs, CECs and OECs, and enabled us to propose that the circulating OEC precursors may consist of viable c-kit^+^ CECs.

## Supporting information

S1 Fig Comparison of the isolated cell fractions between Ficoll vs. RBC treatment. **A**. Overview of the distribution of FACS events in the main regions/populations of interest in Ficoll vs. RBC lysis preparations. Besides differences in the mononuclear vs. non-mononuclear regions, a lower amount of small particles and dead cells was observed in the Ficoll samples. **B**. Percentages of white blood cells in the samples treated with RBC lysis buffer. The distribution matches the expected frequencies. **C**. The standard mononuclear gate used encompasses endothelial cells of all sizes including large OECs and BFA (Bovine Foetal Aortic, from CLS).
(TIF)

S2 Fig Fluorescence minus one (FMO) and isotype controls. (TIF)

S3 Fig Characterization of HPCs. Histograms representative of CD34 (**A**) CD45 (**B**), and c-kit **(C)** levels (measured as median fluorescence intensity, MFI) in different HPC subpopulations from UCB and AB. CD34 levels are higher in CD133^high^ HPCs, independently of c-kit levels. CD45 levels are also positively correlated with CD133 expression, and independent of c-kit levels. The highest c-kit levels are in the CD133^neg^ HPCs. **D**-**E**. Additional details relative to KDR vs. CD146 expression in HPCs (see also Panel D in Fig 3). In (E) for each KDR/CD146-based HPC-subpopulation, the frequency vs. total HPCs and total PBMCs (upper panels) is reported. FSC/SSC (medium panels) and CD45 (lower panels) based identification is also shown to confirm HPC identity. FSC/SSC analysis is from CD34+CD45dim selected HPCs, CD45 analysis is from CD34+ FSC/SSC selected HPCs. (**F**) Additional details relative to CD144 vs. CD146 expression in HPCs, (see also Panel E in Fig 3). The frequency of each subpopulation vs. HPCs and total PBMCs is indicated.
(TIF)

S4 Fig Identification and characterization of CECs. **A**. Identification of CECs using a CD34 vs. CD146 plot, and subsequent CD45 discrimination. The resulting population is pure and not contaminated by HPCs (red, right plot). **B**. Left: KDR expression in CD34/CD146 selected CECs indicated >98% matching. Right: CD105 expression in CD34/CD146 selected CECs shows significant positivity (>50%). **C**. KDR expression is confirmed at mRNA level by RT-PCR in CECs. HPCs are negative.
(TIF)

S5 Fig Images of eEPCs and OECs. **A**. Upper: AB derived eEPCs (total PBMCs, unsorted); lower: UCB derived eEPCs (total PBMCs, unsorted). **B**. eEPCs enriched by sorting out the CD14^+^ fraction from AB-PBMCs (0.5x10^6^ CD14+ events/cm^2^, 1 week culture). **C**. UCB derived OECs with their typical cobblestone morphology (upper panel: early OEC colony; middle panel: confluent OECs).
(TIF)

S6 Fig FACS analysis of HUVECs and OECs. **A**. CD45 and CD14 were invariably 100% negative in HUVECs. CD146, CD144, CD105 were 100% highly expressed and stable. CD133^+^ cells were sporadic (0.8±0.7%). **B**. CD34, c-kit, and KDR were heterogeneously expressed, with higher levels in early (passage 2–4) compared with late cultures (passage ≥10). **C**. Positive correlation between CD34, KDR and c-kit in early HUVEC. **D**. Quantification of CD34, KDR, and c-kit high cells in early vs. late cultures of OECs and HUVECs.
(TIF)

S7 Fig RT-PCR results to angiogenic factors and receptors in the EPC subsets. **A**. Bar diagrams of expressional levels of the genes measured. (mean values and standard deviations). **B**. Differential expressed genes between HPCs and CECs calculated based on two-tailed T-test (Z = standard deviation). **C**. Differential expressed genes between HPCs and OECs calculated based on two-tailed T-test (Z = standard deviation).
(TIF)

S8 Fig Immunohistochemistry of endothelial markers in OECs. vWF = von Willebrand Factor.
(TIF)

S1 Table **Upper Left panel**: FACS settings used. **Upper Right panel**: list of the antibodies used for co-expression analysis in HPCs, CECs and OECs. Details on the staining procedure are reported in Methods. **Lower panel**: primers used for RT-PCR and relative product size. HPRT1 was used low expressed housekeeping gene, B2M as highly expressed housekeeping gene. Additional details are in Methods.
(DOCX)
