# Supplementary figures and images for: Improving the characterization of endothelial progenitor cell subsets by an optimized FACS protocol
Source: PLoS One. 2017 Sep 14;12(9):e0184895. doi: 10.1371/journal.pone.0184895 (PMC5599045; doi:10.1371/journal.pone.0184895)

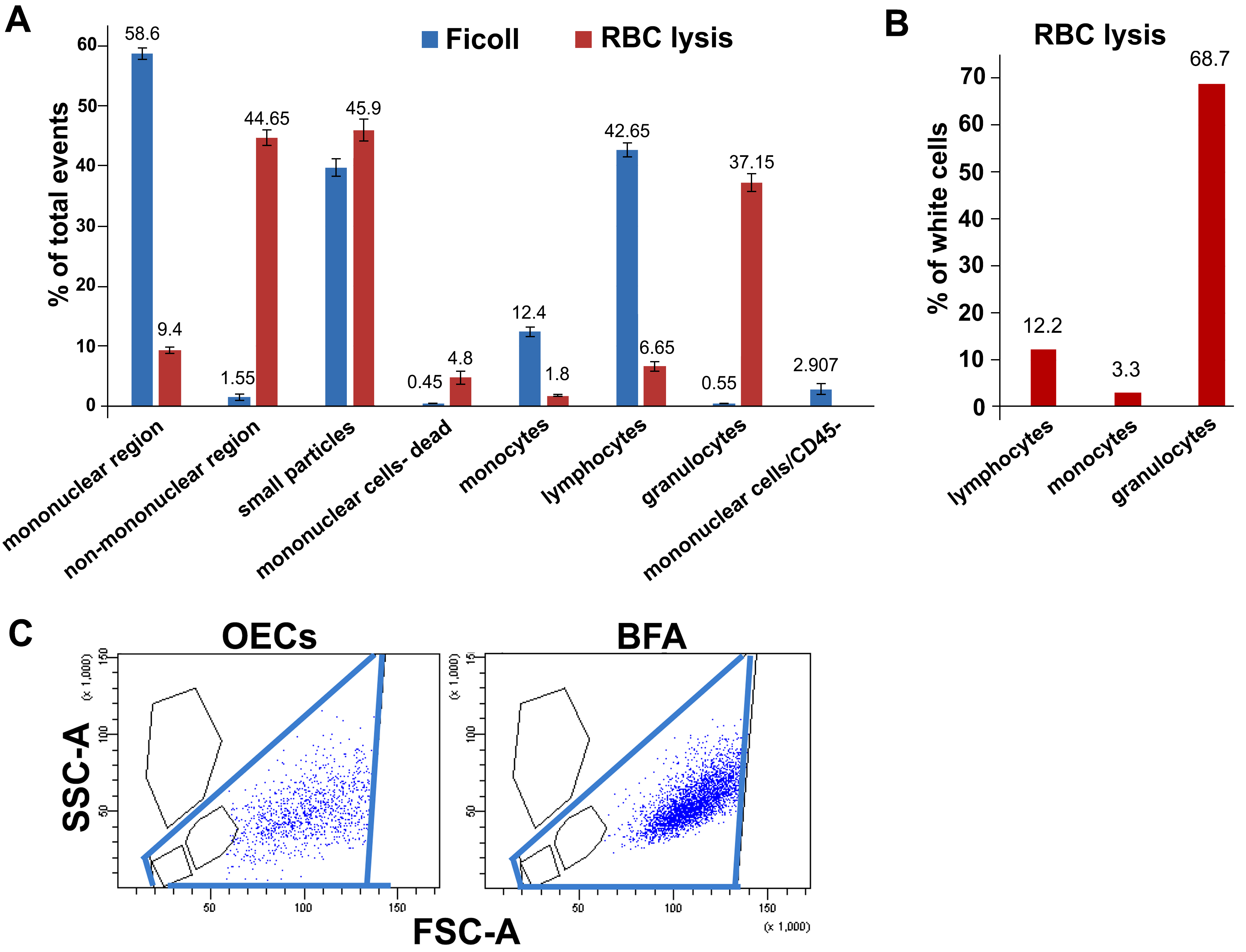

Supplement: S1 Fig — A. Overview of the distribution of FACS events in the main regions/populations of interest in Ficoll vs. RBC lysis preparations. Besides differences in the mononuclear vs. non-mononuclear regions, a lower amount of small particles and dead cells was observed in the Ficoll samples. B. Percentages of white blood cells in the samples treated with RBC lysis buffer. The distribution matches the expected frequencies. C. The standard mononuclear gate used encompasses endothelial cells of all sizes including large OECs and BFA (Bovine Foetal Aortic, from CLS). (TIF) [file pone.0184895.s001.tif]

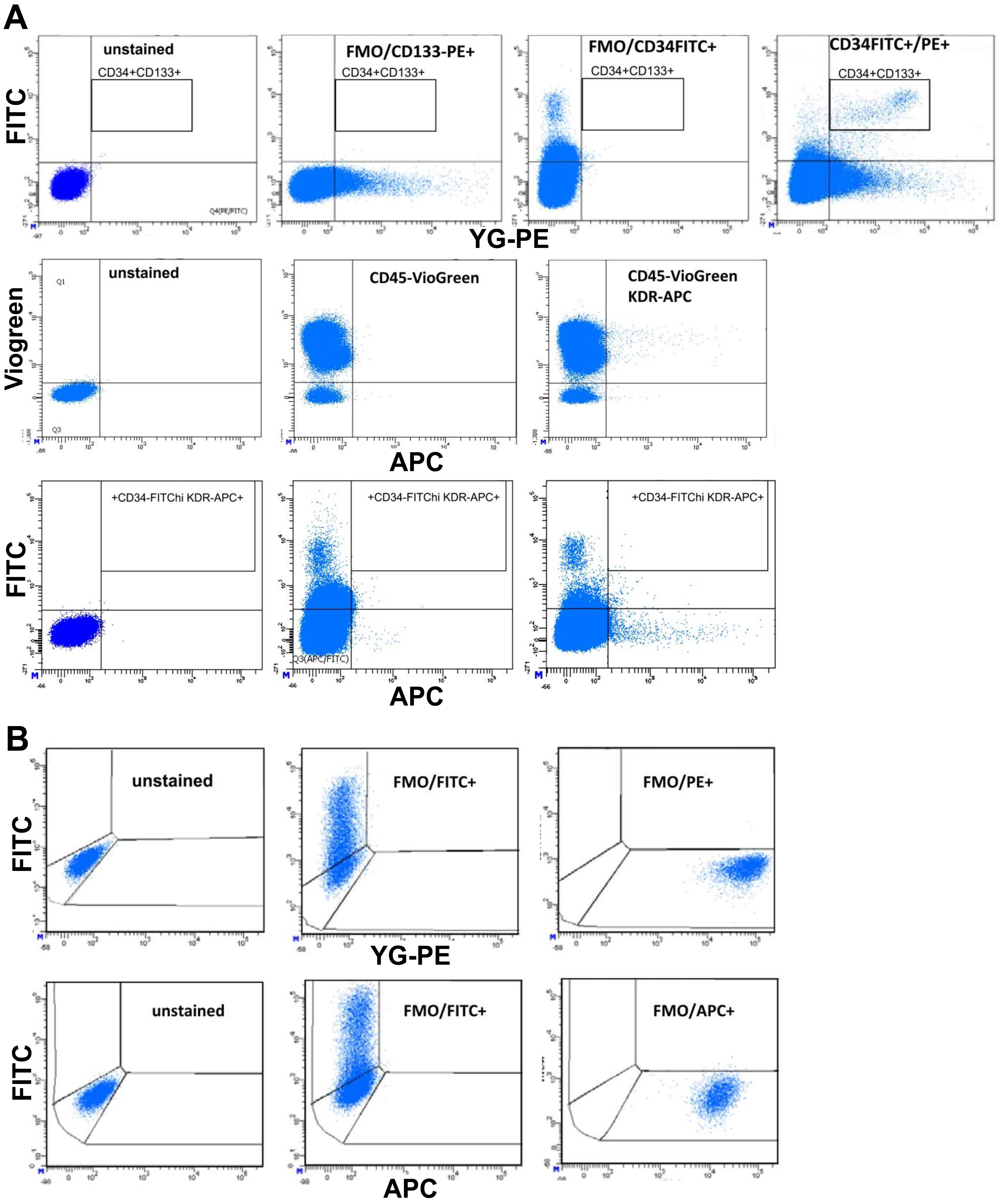

Supplement: S2 Fig — (TIF) [file pone.0184895.s002.tif]

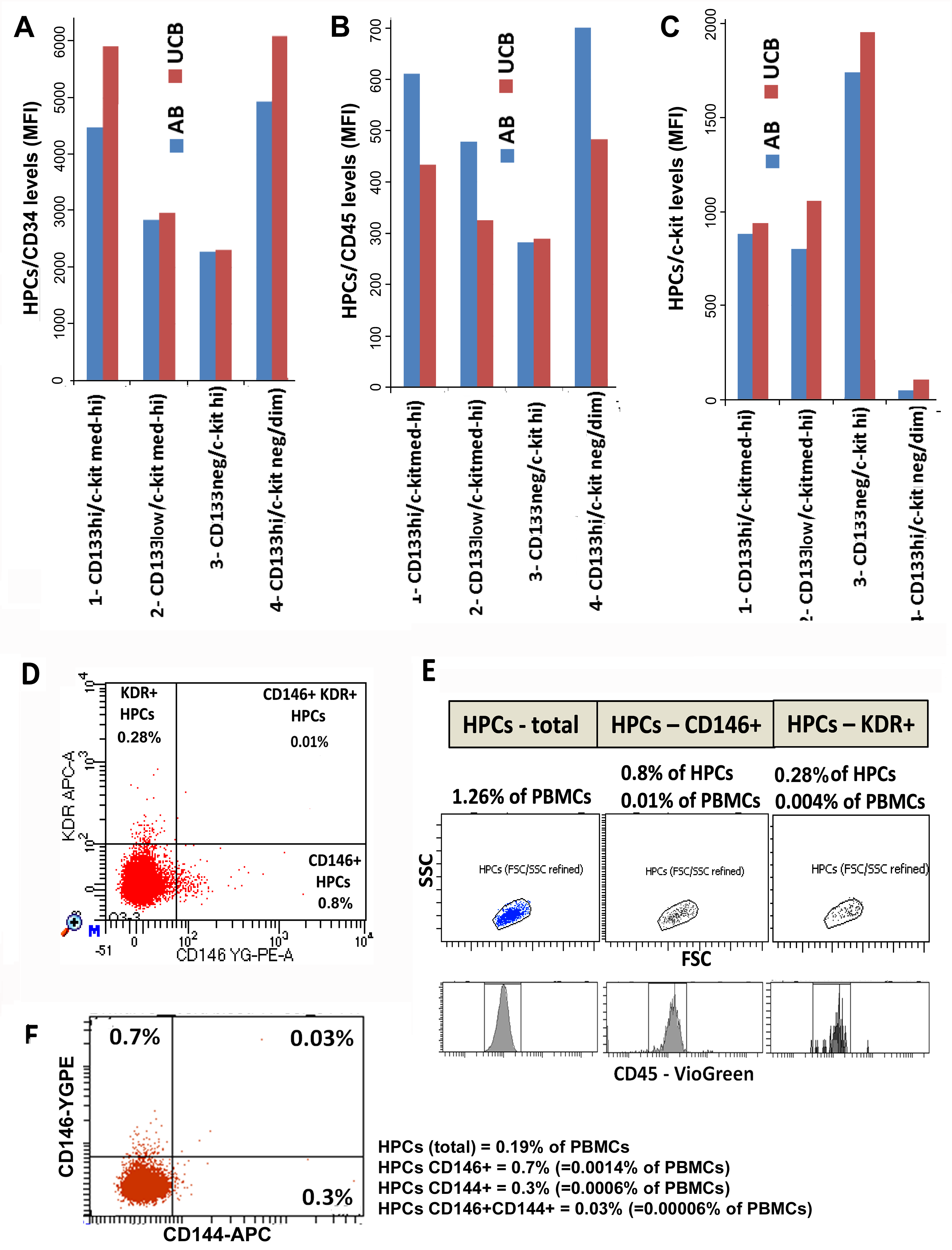

Supplement: S3 Fig — Histograms representative of CD34 (A) CD45 (B), and c-kit (C) levels (measured as median fluorescence intensity, MFI) in different HPC subpopulations from UCB and AB. CD34 levels are higher in CD133high HPCs, independently of c-kit levels. CD45 levels are also positively correlated with CD133 expression, and independent of c-kit levels. The highest c-kit levels are in the CD133neg HPCs. D-E. Additional details relative to KDR vs. CD146 expression in HPCs (see also Panel D in Fig 3). In (E) for each KDR/CD146-based HPC-subpopulation, the frequency vs. total HPCs and total PBMCs (upper panels) is reported. FSC/SSC (medium panels) and CD45 (lower panels) based identification is also shown to confirm HPC identity. FSC/SSC analysis is from CD34+CD45dim selected HPCs, CD45 analysis is from CD34+ FSC/SSC selected HPCs. (F) Additional details relative to CD144 vs. CD146 expression in HPCs, (see also Panel E in Fig 3). The frequency of each subpopulation vs. HPCs and total PBMCs is indicated. (TIF) [file pone.0184895.s003.tif]

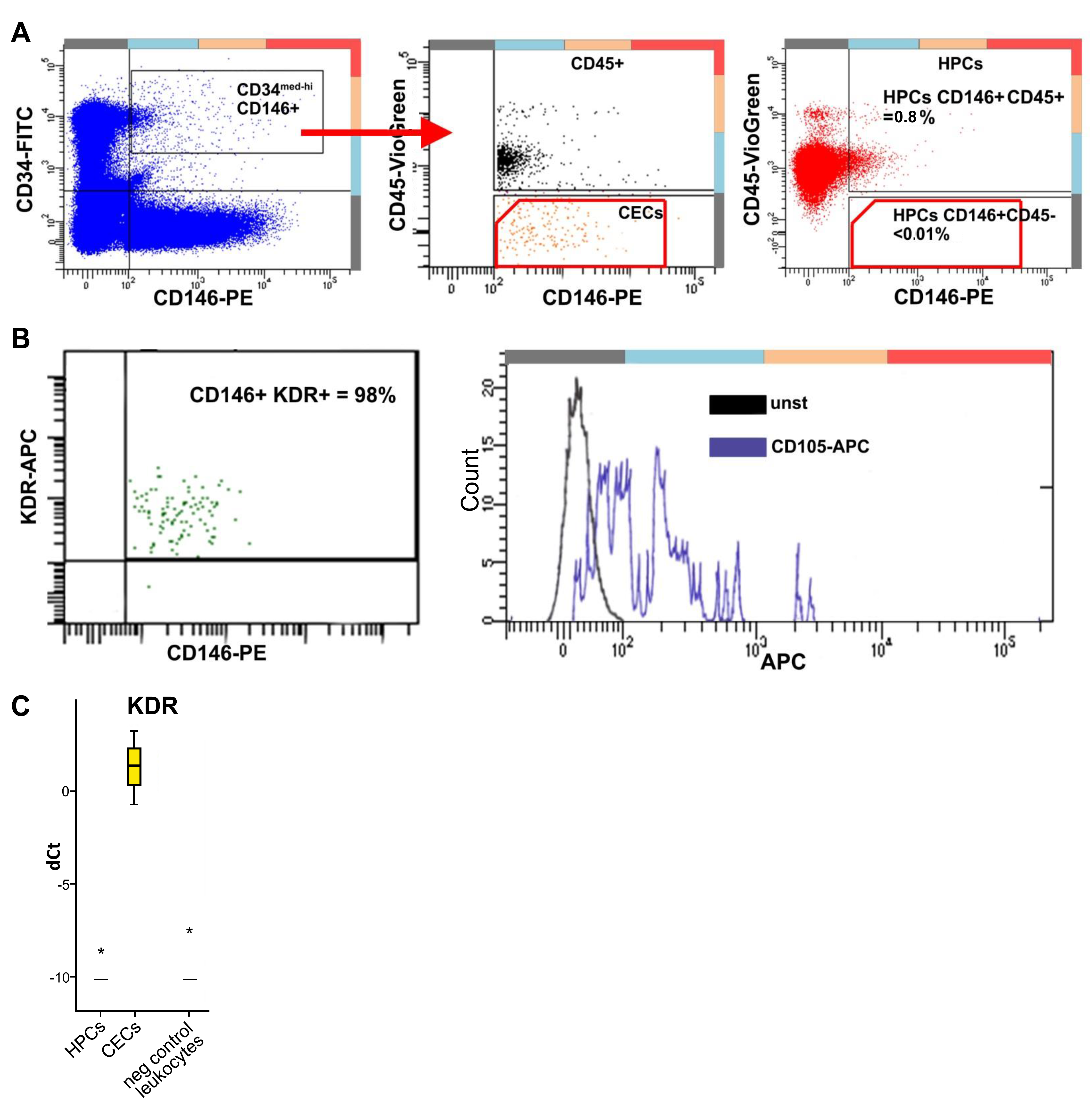

Supplement: S4 Fig — A. Identification of CECs using a CD34 vs. CD146 plot, and subsequent CD45 discrimination. The resulting population is pure and not contaminated by HPCs (red, right plot). B. Left: KDR expression in CD34/CD146 selected CECs indicated >98% matching. Right: CD105 expression in CD34/CD146 selected CECs shows significant positivity (>50%). C. KDR expression is confirmed at mRNA level by RT-PCR in CECs. HPCs are negative. (TIF) [file pone.0184895.s004.tif]

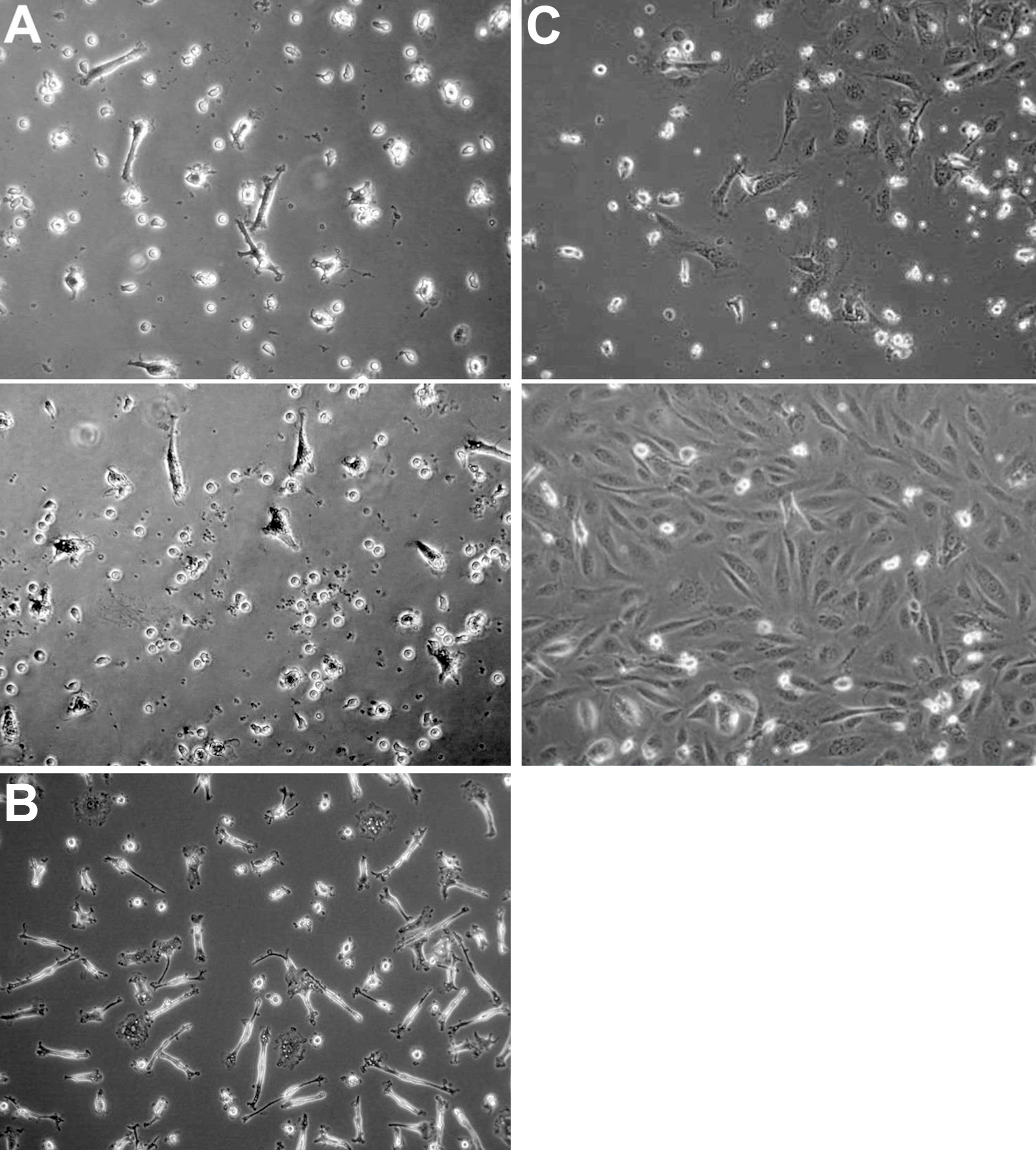

Supplement: S5 Fig — A. Upper: AB derived eEPCs (total PBMCs, unsorted); lower: UCB derived eEPCs (total PBMCs, unsorted). B. eEPCs enriched by sorting out the CD14+ fraction from AB-PBMCs (0.5x10^6^ CD14+ events/cm2, 1 week culture). C. UCB derived OECs with their typical cobblestone morphology (upper panel: early OEC colony; middle panel: confluent OECs). (TIF) [file pone.0184895.s005.tif]

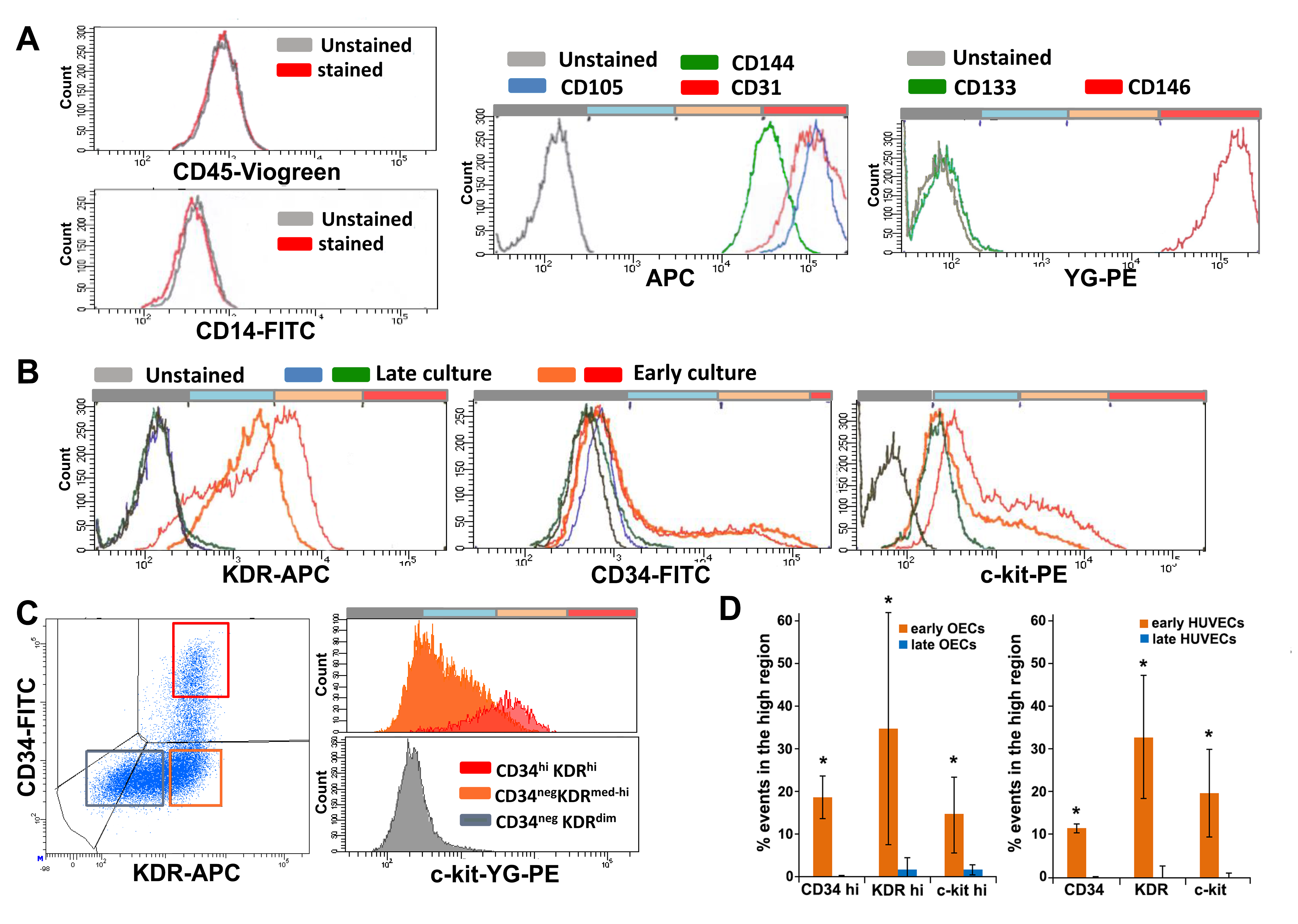

Supplement: S6 Fig — A. CD45 and CD14 were invariably 100% negative in HUVECs. CD146, CD144, CD105 were 100% highly expressed and stable. CD133+ cells were sporadic (0.8±0.7%). B. CD34, c-kit, and KDR were heterogeneously expressed, with higher levels in early (passage 2–4) compared with late cultures (passage ≥10). C. Positive correlation between CD34, KDR and c-kit in early HUVEC. D. Quantification of CD34, KDR, and c-kit high cells in early vs. late cultures of OECs and HUVECs. (TIF) [file pone.0184895.s006.tif]

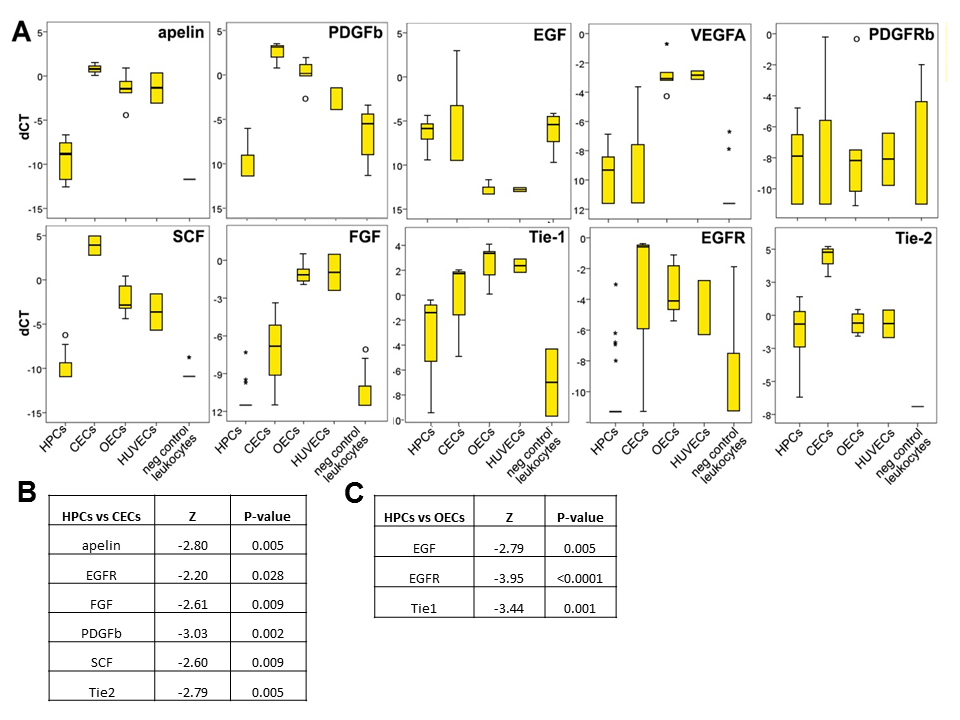

Supplement: S7 Fig — A. Bar diagrams of expressional levels of the genes measured. (mean values and standard deviations). B. Differential expressed genes between HPCs and CECs calculated based on two-tailed T-test (Z = standard deviation). C. Differential expressed genes between HPCs and OECs calculated based on two-tailed T-test (Z = standard deviation). (TIF) [file pone.0184895.s007.tif]

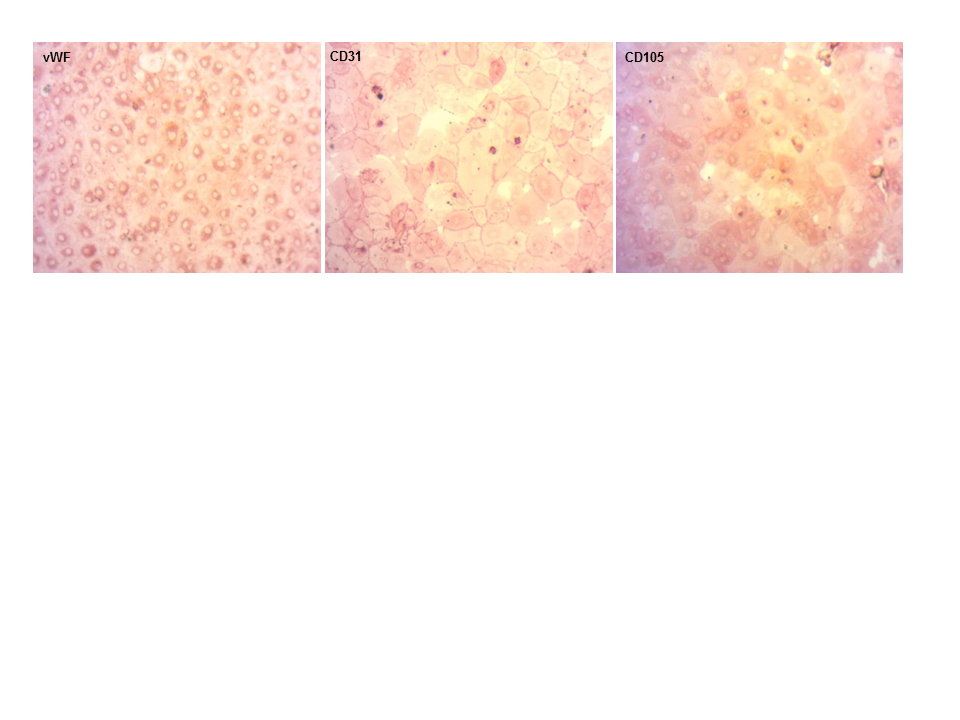

Supplement: S8 Fig — vWF = von Willebrand Factor. (TIF) [file pone.0184895.s008.tif]
