# Supplementary material for: Improving the characterization of endothelial progenitor cell subsets by an optimized FACS protocol
Source: PLoS One. 2017 Sep 14;12(9):e0184895. doi: 10.1371/journal.pone.0184895 (PMC5599045; doi:10.1371/journal.pone.0184895)
Supplement: S1 Table — Upper Left panel: FACS settings used. Upper Right panel: list of the antibodies used for co-expression analysis in HPCs, CECs and OECs. Details on the staining procedure are reported in Methods. Lower panel: primers used for RT-PCR and relative product size. HPRT1 was used low expressed housekeeping gene, B2M as highly expressed housekeeping gene. Additional details are in Methods. (DOCX) [file pone.0184895.s009.docx]

| **BD FACS Aria III Settings** | | | |
| --- | --- | --- | --- |
| **flow cell:** | square |  |  |
| **nozzle:** | 85 or 100µm |  |  |
|  |  |  |  |
| **fluorophore** | **lasers** | **BP filter** | **LP filter** |
| Hoechst/DAPI | 405 nm | 450/40 |  |
| Violet Green | 405 nm | 430/30 | 502 |
| FITC | 488 nm | 430/30 | 502 |
| PE (YG-PE) | 561 nm | 582/15 |  |
| APC | 633 nm | 660/20 |  |
| PerCP-Cy5.5 | 488 nm | 695/40 | 655 |

| **List of antibodies used** | | | |
| --- | --- | --- | --- |
| **Antigen** | **Fluorochrome** | **Company** | **cat. #** |
| CD34 | FITC | Southern Biotech | 9597-02 |
| CD133 | PE | MACS Miltenyi | 130-080-801 |
| KDR | APC | MACS Miltenyi | 130-093-601 |
| CD45 | Viogreen | MACS Miltenyi | 130-096-906 |
| anti-APC | biotin | Biolegend | 408003 |
| streptavidin | APC | BD Pharmingen | 554067 |
| CD144 | APC | eBiosciences | 17-1449-42 |
| CD146 | PE | BD Biosciences | 550315 |
| c-kit (CD117) | APC | MACS Miltenyi | 130-091-733 |
| c-kit (CD117) | PE | MACS Miltenyi | 130-091-734 |
| CD105 | APC | Biolegend | 323208 |
| CD31 | APC | eBioscience | 17-0319-73 |

**
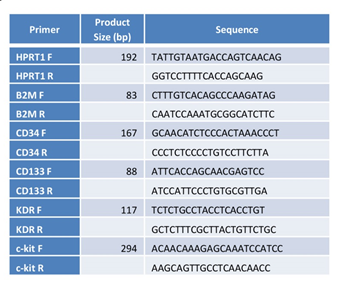
**

**Table S1. Upper Left:** FACS settings used. **Upper Right:** list of the antibodies used in different combinations for co-expression analysis in HPCs, CECs and OECs. Details on the staining procedure are reported in the M&M section. **Lower Table:** primers used for RT-PCR and relative product size. Further details in M&M section.
